# P-1663. Evaluating Peripartum Antimicrobial Use in Intraamniotic Infection Patients

**DOI:** 10.1093/ofid/ofae631.1829

**Published:** 2025-01-29

**Authors:** Grace Pazienza, Lance Schacht, Mattie Jo Thomas, Jennifer Palomo, Sean Stuart, Sarah Withers, Amy H Crockett, Pamela Bailey

**Affiliations:** Prisma Health - University of South Carolina SOM - -, West Columbia, South Carolina; University of South Carolina School of Medicine, Columbia, South Carolina; University of South Carolina SOM, Columbia, South Carolina; Prisma Health - University of South Carolina SOM - -, West Columbia, South Carolina; Prisma Health - University of South Carolina SOM - -, West Columbia, South Carolina; Prisma Health, Greenville, South Carolina; Prisma Health - University of South Carolina SOM, Greenville, South Carolina; Prisma Health Richland - University of South Carolina, Columbia, South Carolina

## Abstract

**Background:**

Intraamniotic infections (IAI, e.g. chorioamnionitis) require urgent antimicrobial administration. These patients may also require group B Streptococcus (GBS) prophylaxis (ppx) or surgical site infection (SSI) ppx. Many of these patients may receive duplicative antimicrobial coverage due to multiple clinical scenarios. We sought to characterize antimicrobial use in these patients, particularly after a clindamycin shortage generated a change to cefoxitin at our institution in June 2023. We sought to characterize the impact of our transition to cefoxitin monotherapy on overall antimicrobial use in patients diagnosed with IAI.

**Table 1.**
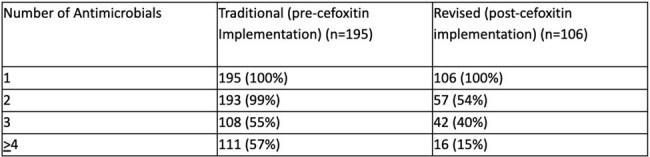
Exposure to Antimicrobials

**Methods:**

A retrospective cohort study was performed. Patients were identified by ICD diagnoses, from February 2016 through October 2023. All charts were reviewed for medication administration as well as appropriate indications. Comparisons were made between the traditional regimen prior to our clindamycin shortage and the revised regimen after cefoxtin was recommended using chi square testing.

**Results:**

We identified 301 patients who received antimicrobials for IAI. 19 patients (6.3%) received antimicrobials for all three indications of IAI, GBS ppx, and SSI ppx.

After cefoxitin-based guidelines implementation, there was a significant difference in the number of antimicrobials to which patients were exposed (p< 0.01, Table 1). There was a significant difference in patients receiving two cephalosporins in the traditional (n=1, 2.8%) and revised (n=34, 97.1%) groups (p< 0.001), and in those receiving two types of penicillin coverage (traditional, n=26, 86.7%) vs revised (n=4, 14.3%) (p=0.008).

Rates of SSI ppx were high for patients with cesarean delivery (n=105/113). A prescribing error was noted with 5 patients undergoing unlabored caesarean deliveries receiving azithromycin. Appropriate prescribing for GBS ppx was also high (n=43/49).

**Conclusion:**

Pregnant persons with IAI are being exposed to significant amounts of antimicrobials during labor and delivery, which was streamlined by our transition to cefoxitin monotherapy for IAI. Many patients received antimicrobials for multiple indications, which should be addressed in guidelines when antimicrobials have overlapping spectrums of activity.

**Disclosures:**

**All Authors**: No reported disclosures

